# The Effectiveness of Eye Movement Desensitization for Post-traumatic Stress Disorder in Indonesia: A Randomized Controlled Trial

**DOI:** 10.3389/fpsyg.2022.845520

**Published:** 2022-04-25

**Authors:** Eka Susanty, Marit Sijbrandij, Wilis Srisayekti, Yusep Suparman, Anja C. Huizink

**Affiliations:** ^1^Faculty of Psychology, Universitas Jenderal Achmad Yani, Cimahi, Indonesia; ^2^Department of Clinical, Neuro- and Developmental Psychology, Faculty of Behavioral and Movement Sciences, Vrije Universiteit Amsterdam, Amsterdam, Netherlands; ^3^Department of General and Experimental Psychology, Faculty of Psychology, Universitas Padjadjaran, Bandung, Indonesia; ^4^Department of Statistics, Faculty of Mathematic and Natural Sciences, Universitas Padjadjaran, Bandung, Indonesia

**Keywords:** eye movements, eye movement desensitization and reprocessing, post-traumati stress disorder, psychological treatments, anxiety, depression, quality of life

## Abstract

**Objective:**

Post-traumatic stress disorder (PTSD) may affect individuals exposed to adversity. Eye Movement Desensitization and Reprocessing (EMDR) is an evidence-based trauma-focused psychotherapy for PTSD. There is still some debate whether the eye movements (EMs) are an effective component of EMDR. The primary aim of this study was to investigate the effectiveness of Eye Movement Desensitization (EMD) treatment in reducing PTSD symptoms compared to a retrieval-only active control condition. We also investigated whether PTSD symptom reduction was associated with reductions in depression and anxiety, and improvements in quality of life.

**Methodology:**

Adult PTSD patients (*n* = 91) were recruited at public psychological services in Jakarta, Bandung and Cimahi, Indonesia. PTSD was diagnosed with the Structured Clinical Interview for DSM-5 disorders (SCID-5). Participants were randomized into: EMD (*n* = 47) or retrieval-only (*n* = 44). EMD consisted of clinical history and treatment planning, preparation, assessment, EMs, closure, whereas retrieval-only consisted of the same elements except EMs. Data were collected at baseline (T0), 1-week post-treatment (T1), 1-month follow-up (T2), and 3-months follow-up (T3). Outcome measures included the PTSD Checklist for DSM-5 (PCL-5), Hopkins Symptoms Checklist-25 (HSCL-25), and the World Health Organization Quality of Life–BREF (WHOQoL–BREF). Data were analyzed with linear mixed model analysis in R Statistics.

**Results:**

Although there were main effects of time indicating reductions for both EMD and retrieval-only in PCL-5 and HSCL-25 scores, and improvements in WHOQoL-BREF scores at T1, T2, and T3, no significant differences in PCL-5, HSCL-25, and WHOQoL-BREF total scores between the EMD and retrieval-only groups at T1, T2, and T3 were found (all group x time interaction *p*’s > 0.005).

**Conclusion:**

Within a clinical sample of PTSD patients in Indonesia, both EMD and retrieval-only was associated with reductions in symptoms of PTSD, anxiety and depression, and improvements in quality of life, although EMs did not add to the efficacy of the treatments. Further research to examine the underlying mechanisms of EMDR’s effective treatment elements in clinical samples is needed.

**Clinical Trial Registration:**

[www.ClinicalTrials.gov], identifier [ISRCTN55239132].

## Introduction

Post-traumatic stress disorder (PTSD) is a common mental disorder that may occur after exposure to a traumatic event, such as a natural disaster, a terrorist attack, a serious accident, or a physical or sexual assault in adult- or childhood. Most people do not experience any PTSD symptoms or experience initial symptoms in the first few days or weeks after the event that reduce naturally. However, some people will continue to experience intrusion symptoms, avoidance, negative alteration in cognition and mood, and alterations in arousal and reactivity, and develop PTSD (American American Psychiatric Association [APA], 2013). PTSD symptoms may significantly impair the ability to function in social and family life, negatively affect quality of life (Fu et al., 2013) and reduce overall psychological and physical health (McFarlane, 2010).

PTSD prevalence rates vary widely, depending on various risk factors, including sociodemographic and background factors, trauma type, social support or the intensity of the acute response to the traumatic event (Neria and Galea, 2008; Farooqui et al., 2017; Marthoenis et al., 2019). A systematic review revealed that the prevalence of PTSD in the first year after exposure among survivors of a natural disaster ranges between 30 and 40%; the prevalence range of PTSD among rescue workers was 10 and 20%, as compared to a PTSD prevalence in the general population ranging between 5 and 10% (Neria and Galea, 2008). In Indonesia, where the current study was conducted, the overall PTSD rate in 3–6 years after major natural disasters in Sumatera and Java was 20.9% (Downs et al., 2017).

Several psychotherapies have been proven to be effective in treating PTSD. Trauma-focused cognitive-behavioral therapy (TF-CBT), cognitive processing therapy (CPT) and Eye Movement Desensitization and Reprocessing (EMDR) have the strongest evidence base (Cusack et al., 2016; Lancaster et al., 2016; Tran and Gregor, 2016), and are the most recommended treatments for adults with PTSD).

EMDR is a form of psychotherapy developed by Shapiro (2002, 2007), that is widely used to treat PTSD patients. A specific aspect of EMDR is bilateral stimulation during retrieval of the traumatic memory. EMDR engages a person in imaginal exposure to trauma, while simultaneously performing saccadic eye movements. As such, patients are required to divide their attention between bilateral stimulation and the retrieval of traumatic memories (Shapiro and Maxfield, 2002).

Several explanations for the working mechanism of eye movements in EMDR have been proposed. For example, it has been suggested that Ems elicit an orienting response (Sack et al., 2008; Schubert et al., 2011). According to this theoretical model, EM activates an “investigatory reflex,” in which first an alert response occurs, and next a reflexive pause produces a relaxation sensation that inhibits negative affect related to the traumatic memory (Cusack et al., 2016). Furthermore, it increases alertness and facilitates exploratory behavior, which is assumed to evoke more flexible and efficient cognitive processes (Kuiken et al., 2001). Another theoretical framework is the Adaptive Information Processing (AIP) model, which posits that the primary source of psychopathology is the presence of traumatic memories inadequately processed by the brain (Hase et al., 2017). According to this model, the “re-processing,” promotes integration into adaptive memory networks, leading to a resolution of symptoms, and enabling learning (Solomon and Shapiro, 2008; Hase et al., 2017).

Most empirical support was found for the Working Memory explanation for the working mechanism of EMDR, stating that when the two tasks of recalling unpleasant memories and EMs are performed simultaneously, the working memory becomes less efficient as EMs use up processing resources (van den Hout and Engelhard, 2012). This may deteriorate the quality of the trauma image even upon reconsolidation into the episodic memory (Gunter and Bodner, 2008; Maxfield et al., 2008; Engelhard et al., 2011). The role of EMs as part of EMDR is assumed to cause unpleasant memories to become less vivid and less unpleasant and thus lead to overall improvements in terms of PTSD symptoms (Gunter and Bodner, 2008; van den Hout and Engelhard, 2012; Jeffries and Davis, 2013).

Support for the Working Memory (WM) model has been found in several experimental studies in healthy participants. Mertens et al. (2021) conducted a meta-analysis of 45 studies to evaluate the Working Memory model specifically on dual-task intervention studies within the laboratory to attenuate emotional memories and intrusive mental images. The authors concluded that taxing the working memory by performing dual tasks such as EMs or counting reduced vividness and emotionality of intrusive mental image and emotional memories (Mertens et al., 2021). These results are in line with another meta-analysis on the effect of dual-task interventions, which also indicated that EMs yielded a stronger overall vividness reduction than alternative dual tasks (Houben et al., 2020).

In addition, a number of studies in PTSD patients were carried out to investigate the additive benefit of EMs. A small study of Thomaes et al. (2016) in eight patients with PTSD compared recalling the traumatic memory with EMs to a “recalling-only” condition within a cross-over experimental design. The results showed that subjective vividness and emotionality of the traumatic memory did not change significantly over time in both conditions. A study of Sack et al. (2016) in 139 patients with PTSD, compared regular EMDR with either EMDR fixating on the therapist’s non-moving hand or with an exposure only condition. It was found that both EMDR conditions led to stronger reductions in PTSD symptoms than exposure only, but there were no differences between following a moving hand vs. a non-moving hand (Sack et al., 2016). A meta-analysis by Lee and Cuijpers (2013) compared the effects of EMDR therapy with eye movements to interventions with a similar procedure but without eye movements (exposure only) in a meta-analysis across 14 studies. They found that EMs in EMDR had a moderate and significant beneficial additive effect. However, the quality of the majority of included studies in this meta-analysis (Lee and Cuijpers, 2013) was low due to, for instance, a lack of adequately handling incomplete data or not describing adequate sequence generation used to randomize the participants in the different conditions. Furthermore, previous clinical studies comparing the effect of EMDR therapy with eye movements to those of EMDR without the eye movement are hampered by small sample sizes (i.e., largest sample size *N* = 25; Devilly et al., 1998), no randomization (Devilly et al., 1998), or lack of therapist training (Renfrey and Richard Spates, 1994). The low quality of research involving EMDR in previous studies has prevented us from building robust conclusions regarding the role of EM in the EMDR procedure when evaluated in patient samples. In a recent meta-analysis, Cuijpers et al. (2020) examined the effect of EMDR on PTSD and other mental health problems. The study found five dismantling studies specific on PTSD, in which full EMDR was compared with EMDR in which one component was removed (e.g., Sack et al., 2016). The results showed that the pooled effect size of full EMDR vs. partial EMDR was non-significant. Three of these studies had a high risk of bias (Cuijpers et al., 2020). Despite some indications showing that EMs may add to EMDR’s effectiveness, until now dismantling studies are very scarce, and there is still debate around the working mechanism of EMDR.

Our study aimed to improve the methodology by including a relatively large sample, randomizing the participants, trained therapists, which would potentially increase the fidelity of the intervention, and comparison conditions that allowed us to test for the specific effect of EMs. In the current study, we focus on EMDR, and more specifically, on the first part of this treatment: Eye Movement Desensitization (EMD) and compare it with retrieval-only in terms of its effectiveness in reducing symptoms of PTSD, anxiety, and depression, and improving quality of life in PTSD patients. We did not include the installation phase of the regular EMDR treatment, since it has been suggested that this phase may render a positive image or thought less vivid and positive, which would be counter-productive in improving mental health (van den Hout and Engelhard, 2012). We compared EMD with a retrieval-only comparison group, which received the same treatment as the EMD group participants, except that during desensitization, no eye movements were performed during retrieval of the traumatic memory.

## Materials and Methods

### Study Design

We conducted an RCT to test the effectiveness of EMD vs. a retrieval-only control condition, which consists of the EMD protocol without EMs. The study protocol was approved by the Research Ethics Committee of Universitas Padjadjaran Bandung on 2 July 2018 (Document number: 3 35/UN6.KEP/EC/2018). For a detailed description of the study protocol (see Susanty et al., 2021b). We adhered to the CONSORT statement and referred to the CONSORT checklist (Supplementary Appendix A).

### Participants

We approached outpatients or inpatients in one of the participating centers in Indonesia: (1) the “Pulih” clinic in Jakarta, (2) the “Unisba psychology service” in Bandung and (3) the “Unjani crisis center” in Cimahi. Inclusion criteria were: (1) increased levels of PTSD as indicated by a PTSD Checklist for DSM-5 (PCL-5) cut-off score of 33 or higher (Bovin et al., 2016); (2) a diagnosis of PTSD as diagnosed with the Structured Clinical Interview for DSM-5 disorders (SCID-5); and (3) age of 18 years or older. Exclusion criteria (determined by chart review of the SCID-5) were: (1) current or previous psychotic disorder; (2) current substance use disorder; (3) acute suicidality; and (4) current organic disorder, i.e., epilepsy, brain damage.

We based the power calculations on expecting a significant difference between the two treatment arms on the primary outcome, which was a stress measure outcome as described in the study protocol (Susanty et al., 2021a). In order to detect a difference with an expected effect size of *d* = 0.4 (see Lee and Cuijpers, 2013), power calculations suggested a minimum sample size of 41 participants per group anticipating 25% drop out at follow-up, 110 participants (55 per group) were aimed for.

### Study Procedures

A trained assessor provided information about the purpose of the study, including the study rationale, risks and safety, benefits, and their right to withdraw from the study at any time without consequences. Oral and written informed consent was obtained from all participants. The trained assessor continued the baseline assessment by administering the Hopkins Symptoms Checklist-25 (HSCL-25), and the World Health Organization Quality of Life (WHOQoL-BREF).

After all baseline assessments were completed, participants were allocated on a 1:1 ratio using block randomization using the Castor data management software^1^ into one of two conditions: (1) EMD or (2) retrieval-only control. Block sizes of 4, 6, and 8 were applied to allocate the participants. The time span between T0 and the first intervention session was approximately 1 week. Upon randomization, participants received 4–6 EMD or retrieval-only weekly therapy sessions. Participants completed the self-report measures at 1-week post-treatment (T1), at 1 month (T2) and 3 months (T3) after treatment completion. The assessors were blinded to the study conditions.

### Treatment Conditions

Both treatment conditions were delivered individually and in-person by experienced psychotherapists with at least 1 year experience in treating PTSD patients. Eight therapists were recruited through colleagues from the Clinical Psychologist Association (IPK) in Indonesia. Therapists received extensive training in EMD and retrieval-only, including case practices with supervision and ongoing weekly supervision by an accredited EMDR supervisor throughout the duration of the study. Sessions were video recorded for supervision purposes. The therapists were responsible for delivering the treatment conditions. A minimum of four to a maximum of six sessions were given in order to minimize heterogeneity. If the participant reached a score of 0 or 1 at the fourth or fifth session, the therapy was ended. At the end of the sixth session, the therapy was always ended. Each EMD session lasted 45–60 min.

### Eye Movement Desensitization Treatment Procedure

In this study, the procedures of EMD were carried out in line with the standard EMDR protocol (Shapiro and Maxfield, 2002). Furthermore, we decided to omit the installation phase in both study groups. It has been suggested that the installation phase may be counter-effective since performing eye movements when retrieving a positive cognition or image (as done in the installation phase) may render that positive image less vivid and positive (van den Hout and Engelhard, 2012). In this study, EMD consists of the following steps: (***1) Client history*** **and** ***treatment planning***: obtaining information regarding the clients’ clinical condition, including intrusive emotions and physical sensations. (***2) Preparation***: building a therapeutic bond with the client, providing an explanation of the EMDR process and its possible effects. (***3) Assessment***: identification of the target visual image of the traumatic memory and associated negative emotions. The participant then rates the intensity of the negative emotions on the 0–10 SUD scale). (***4) Desensitization***: clients were asked to focus on target traumatic events, while focusing their eyes on the therapist’s finger that moves from left to right and back in the participant’s visual field. The therapist conducted the EMs bilateral stimulation for 24 cycles several times. Before and after the desensitization phase, the client was asked to rate Subjective Units of Distress (SUD)—a scale ranging from 0 to 10 to measure the subjective distress that the client feels (Shapiro and Forrest, 2004). This phase ended if the SUD scores reached 0 or 1. Next, participants were instructed to scan their body until any sensation of tension disappears. (*5**) Closure***: the session ends, and the stabilization techniques and relaxation exercises were reviewed. Sessions 2–4 started with a reevaluation of the patient’s progress and SUD scores of the target events to guide the choice of continuing the therapy with the target traumatic event or choosing a new event.

### Retrieval-Only Condition (Control)

Control participants received the same treatment as the participants in the EMD group, except that during phase (4) Desensitization, no eye movements were performed during retrieval of the traumatic memory.

### Measures

The SCID-5 is a well-established structured clinical interview to diagnose all DSM-5 Axis I disorders, including PTSD (Glasofer et al., 2015). We have used the Bahasa Indonesian version of SCID-5 (Arjadi et al., 2018). We administered three modules of the Indonesian version of the SCID-5 (Arjadi et al., 2018) during screening: the Trauma and Stressor-Related Disorders, Psychotic and Associated Symptoms, and Substance Use Disorders Modules.

The PCL-5 is a 20-item self-report measure that assesses the DSM-5 criteria for PTSD symptoms experienced in the last month. The participants rated their PTSD symptoms on a scale from 0 to 4 [“not at all (0)” to “extremely (4)”]. Items are summed to provide a total severity score and total score range from 0 to 80, with higher scores indicating higher symptoms severity. The PCL-5 showed good psychometric properties (Blevins et al., 2015). The Indonesian version of the PCL-5 has been proven a valid and reliable questionnaire in Indonesia. The internal consistency coefficient (Cronbach’s alpha) for the total scale of PCL-5 was 0.93. Cronbach’s alpha ranged between 0.75 and 0.85 for different subscales (Asti, 2015).

### Other Measures

The HSCL-25 is a 25 item self-report measure that assesses anxiety and depression symptoms. The HSCL-25 consists of two parts: anxiety symptoms (10 items) and depression (15 items). Symptoms are scored on a five-point scale varying from “not at all (0)” to “extremely (4).” Total scores range from 0 to 100, ranging from 0 to 40 for anxiety and 0–60 for depression, with higher scores indicating more symptoms. The HSCL-25 has been translated and culturally adapted for use in Indonesia (Turnip and Hauff, 2007), and has been found to be reliable and valid across a variety of cultural groups, including Indonesia (Larson-stoa et al., 2015).

The WHOQoL-BREF consists of 26 items, two of which measure overall quality of life and general health (World Health Organization [WHO], 1996). The other 24 items are divided into four domains: physical, psychological, social relationships and environmental domains during the past 4 weeks (Purba et al., 2018). Domain scores are scaled in a positive direction (1 = not at all, 2 = not much, 3 = moderately, 4 = a great deal, 5 = completely). The scores are transformed into a linear scale between 0 and 100, with lower scores indicating lower levels of quality of life. The Indonesian WHOQoL-BREF has shown adequate psychometric properties (Purba et al., 2018). Cronbach’s alpha ranged between 0.41 and 0.77 for the different subscales. The lowest Cronbach’s alpha was found for the social relationships domain (<0.5) (Salim et al., 2007).

### Data Analysis

Baseline sociodemographic data and outcomes of interest were first compared between treatment conditions using chi-square tests and independent-samples *t*-test in SPSS version 26. We also compared baseline demographic and clinical characteristics between patients who dropped out vs. those who did not drop out at T1, T2, and T3. We used logistic regression analysis to evaluate dropout at T1, T2, and T3, based on the characteristics and treatments given in the experiment or treatments outside the experiment.

We analyzed the effects of EMD vs. retrieval only on symptoms of PTSD, anxiety, and depression, and quality of life using linear mixed models in R versions 3.6.1 using the “nlme” package (Linear and Non-linear Mixed Effects Models. R package version 3.1-152), with a random effects model. We included time, condition (EMD vs. retrieval only), and time by condition. All outcomes were reported as unstandardized regression coefficients. In all analyses, a treatment x time interaction term represented the effect of EMD and retrieval-only interventions on the outcome variables over time. In our trial (see Susanty et al., 2021a) we planned 11 tests in total to examine our primary and secondary outcomes as were discussed in our protocol paper (Susanty et al., 2021a). These outcomes included the measures as described in this paper (PTSD Checklist for DSM-5 (PCL-5), Hopkins Symptoms Checklist-25 (HSCL-25), and the brief version of World Health Organization Quality of Life (WHOQoL BREF) and other measures, related to stress (e.g., Heart Rate Variability, Heart Rate, cortisol) and cognitive outcomes such as Digit Span (WAIS-IV), California Verbal Learning Test (CVLT) and Trail Making Test (TMT). To correct for multiple testing within our trial, we used *post-hoc* test by applying Bonferroni correction considering 11 planned tests (alpha level was 0.05/11 = 0.005). Intention to treat (ITT) analysis was conducted using the data of all randomized participants, with missing data imputed using the regression method for participants who did not complete the T2 and/or the T3. Second, the per-protocol analyses were conducted using only the data of participants who completed the treatment (at least 4 sessions).

## Results

### Participants

**FIGURE 1:**
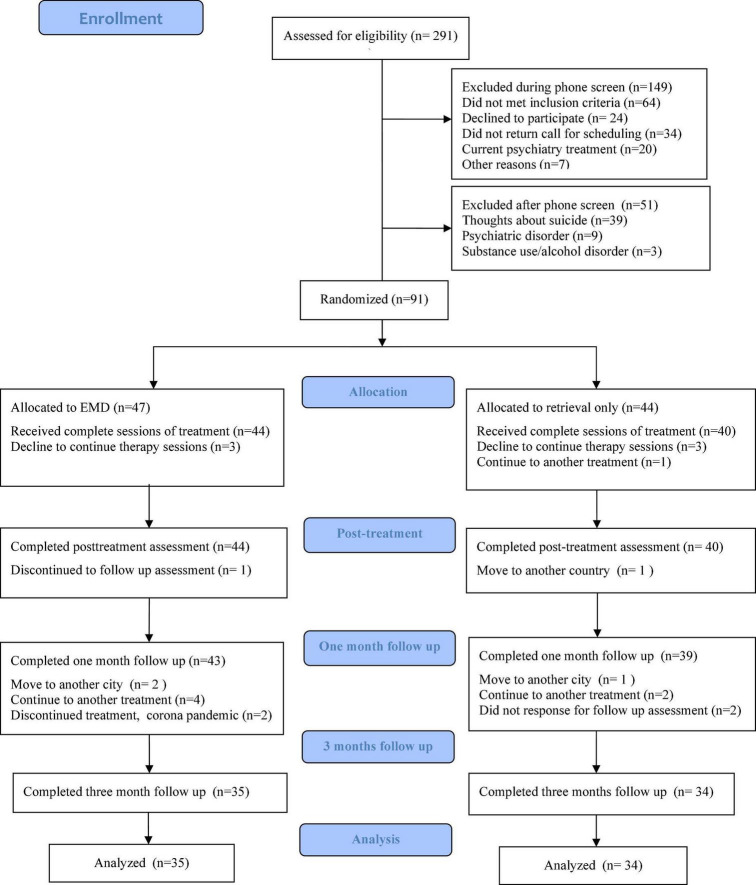
CONSORT flow diagram.

The enrolment and flow of participants throughout the study is summarized in Figure 1. Of the 291 participants approached, 91 (31.3%) agreed to participate in the study. We excluded 149 candidates after the first round of screening, due to the following reasons: 64 did not meet the inclusion criteria, 24 declined to participate, 34 did not return the call for scheduling, 20 had current psychiatric comorbidities and 7 had other reasons. Then, 51 candidates were excluded after the second round of screening, with the following reasons: 39 had thoughts about suicide, three had substance use/alcohol disorder, and nine had another psychiatric disorder, of whom two had bipolar disorder, two had paranoid personality disorder, and five had hallucination symptoms. We refer the individuals who had thought about suicide to another psychological crisis center for further help. Further, seven participants, who were randomly assigned to the intervention (EMD group) or control (retrieval only group), withdrew after randomization and baseline assessment; five of these seven participants declined to continue the therapy sessions, one participant failed to be contacted, and one participant switched to another treatment.

Ninety-one participants were assigned to EMD (*n* = 47) or retrieval-only (*n* = 44). At T1, 93.6% (44/47) of the EMD group and 90.9% (40/44) of the retrieval-only group completed their 1-week follow-up after treatment. At T2, 91.5% (43/47) of the EMD group and 88.6% (39/44) of the retrieval-only group completed the 1-month assessment after treatment. At T3, 74.5% (35/47) of the EMD group and 77.3% (34/44) of the retrieval-only group completed the 3-months follow-up assessment. Additionally, at T1 and T2, the patients who dropped out did not differ significantly from the patients who completed the assessments in terms of medical history and sociodemographic background. At T3, participants who received previous treatment for PTSD were more likely to dropout at T3 than those who did not (OR 19.4; 95% CI 1.11–338.26). Meanwhile, subjects without prior or concurrent medical histories were more likely to drop out (OR 4.1, 95% CI 1.10–15.38).

Descriptive statistics for baseline variables and sociodemographic characteristics by group are displayed in Table 1. There were no significant differences between the EMD and retrieval-only groups in demographics, symptom measures and quality of life at baseline (see Table 1). Most of the participants were females: 89.4% (42/47) of the EMD and 93.2% (41/44) of the retrieval-only group, and more than half of participants had experienced domestic violence: 57.4% (27/47) of the EMD, and 56.8% (25/44) of the retrieval-only group.

**TABLE 1:** Baseline characteristics of participants.

Variable	Total *N* = 91	Retrieval only (*n* = 44)	EMD (*n* = 47)	χ^2^ or *t*	*p*-value*
**Age, mean (*SD*)**		24.66 (5.51)	26.15 (6.81)	–1.14	0.26
**Sex, *n* (%)**				0.41	0.52
Male	8 (8.8)	3 (6.8)	5 (10.6)		
Female	83 (91.2)	41 (93.2)	42 (89.4)		
**Education, *n* (%)**				3.98	0.26
High school	33 (36.6)	18 (40.9)	15 (31.9)		
College	5 (5.5)	4 (9.1)	1 (2.1)		
Bachelor	49 (53.8)	21 (47.7)	28 (59.6)		
Master	4 (4.4)	1 (2.3)	3 (6.4)		
**Work, *n* (%)**				0.53	0.77
Unemployed	47 (51.6)	21 (47.7)	26 (55.3)		
Public sector	2 (2.2)	1 (2.3)	1 (2.1)		
Private sector	42 (46.2)	22 (50.0)	20 (42.6)		
**Marital status, *n* (%)**				0.41	0.38
Unmarried	69 (75.8)	33 (75)	36 (76.6)		
Married	21 (23.1)	10 (22.7)	11 (23.4)		
Divorced	1 (1.1)	1 (2.3)	0 (0)		
**Trauma type, *n* (%)**				1.29	0.53
Domestic violence	52 (57.1)	25 (56.8)	27 (57.4)		
Sexual abuse	13 (14.3)	8 (18.2)	5 (10.6)		
Other	26 (28.6)	11 (25)	15 (31.9)		
**Previous mental health treatment**				0.82	0.05
No previous treatment	59 (64.8)	28 (63.6)	31 (66)		
Previous treatment	32 (35.2)	16 (36.4)	16 (34)		
**Medical history**				0.41	0.69
No previous disease	56 (61.5)	29 (66)	27 (57.4)		
Previous disease hospitalization	35 (38.5)	15 (34)	20 (42.6)		
**PCL-5, mean (*SD*)**					
PCL-5 total	58.41 (9.36)	57.93 (9.07)	58.85 (9.68)	–0.47	0.64
Intrusive	15.46 (2.93)	15.48 (3.05)	14.45 (2.85)	–0.05	0.96
Avoidance	6.09 (1.62)	5.86 (1.73)	6.30 (1.49)	–1.28	0.20
Thinking and mood	20.11 (4.59)	19.77 (4.69)	20.43 (4.52)	–0.67	0.50
Arousal and reactivity	16.75 (3.52)	16.82 (3.16)	16.68 (3.87)	–0.19	0.85
**HSCL-25, mean (*SD*)**					
HSCL-25 total	69.25 (17.46)	67.68 (17.17)	70.78 (17.80)	–0.84	0.41
Anxiety	27.83 (7.68)	27.09 (7.56)	28.56 (7.82)	–0.90	0.37
Depression	41.47 (11.61)	40.59 (11.78)	42.33 (11.50)	–0.71	0.48
**WHOQoL, mean (*SD*)**					
WHOQoL total	35.25 (5.83)	35.00 (6.13)	35.49 (5.58)	–0.94	0.50
Physical health	6.80 (1.52)	6.91 (1.44)	6.69(1.59	0.68	0.61
Psychological health	7.40 (1.58)	7.32 (1.47)	7.49 (1.69)	–0.51	0.41
Social relationship	14.29 (3.83)	13.95 (3.92)	14.62 (3.76)	–0.82	0.72
Environment	6.75 (1.67)	6.82 (1.69)	6.69 (1.68)	0.36	0.69

*Significance, p < 0.05.

*Chi-square test for nominal variables and independent samples t-tests for continuous variables.*

*Previous treatment; participants who had counseling, psychotherapy, hypnotherapy.*

*Previous disease hospitalization; participants who had dengue fever, typhoid, bronchitis, mammae tumor, ovarium tumor, gastritis, thalassemia, appendicitis, HIV.*

*EMD, Eye Movement Desensitization; HSCL-25, the Hopkins Symptom Checklist- 25; PCL-5, PTSD Checklist for DSM-5; WHOQoL-BREF, World Health Organization Quality of Life Scale; SD, Standard Deviation.*

### Treatment Effects

#### Post-traumatic Stress Disorder Symptoms

ITT analysis of the estimates for the effect of group, time, and group-time interactions for PCL-5, HSCL-25, and WHOQoL-BREF are presented in Supplementary Appendix B. There was main effect of time for PCL-5 total scores. Results indicated a significant reduction in PCL-5 total scores from baseline to T3 for both groups (*p* < 0.001). *Post-hoc* tests showed PCL-5 total scores were lower at T1, T2, and T3 than T0 in both groups (Bonferroni-adjusted *p* < 0.005). For both groups, PCL-5 total scores dropped by a clinically significant 23.10 points at 3-months after treatment (T3; *p* < 0.001). We found that there was no effect of group (*p* = 0.92) nor of group-time interactions (*p* = 0.25) on the PCL-5 scores. Thus, no significant differences between the EMD and retrieval-only group were found from baseline to T3 on PCL-5 total scores. Furthermore, there was no significant difference between retrieval-only and EMD groups on any of the PCL-5 subscales. Per-protocol analysis of estimates for the effect of group, time, and group-time interaction for PCL-5 indicated similar results (Supplementary Appendix C).

Linear mixed models (LMM) in the intention to-treat sample for the PCL-5 total score showed no significant difference between retrieval-only and EMD groups at T1 [*M* (*SE*) 29.20 (5.20) vs. 26.60 (5.12), 95% Cl −5.73 to 10.92, *p* = 0.54] or at T2 [*M* (*SE*) 28.30 (5.20) vs. 25.70 (5.12), 95% CI −5.69 to 10.97, *p* = 0.53] or at T3 [*M* (*SE*) 38.30 (5.20) vs. 33.90 (5.12), 95% CI −3.95 to 12.7, *p* = 0.30] (Table 2). Per-protocol analyses for PCL-5 indicated similar results (Supplementary Appendix D).

**TABLE 2:** Summary statistics and results from mixed-model analysis for symptoms of PTSD, anxiety, depression s and quality of life (Intention-to-Treat sample, *N* = 91).

Outcomes	Measurement time	Mean (*S*D)				Mean difference (95% confidence interval)	*p*-value*
		
		Retrieval only		EMD			
PCL-5 total	T1	29.20	5.20	26.60	5.12	2.6(−5.73*to*10.92)	0.54
	T2	28.30	5.20	25.70	5.12	2.64(−5.69*to*10.97)	0.53
	T3	38.30	5.20	33.90	5.12	4.38(−3.95*to*12.7)	0.30
PCL-5 intrusion	T1	7.08	1.10	6.55	1.09	0.53(−1.24*to*2.31)	0.55
	T2	6.85	1.10	5.82	1.09	1.03(−0.74*to*2.81)	0.25
	T3	5.78	1.10	5.42	1.09	0.37(−1.41*to*2.14)	0.68
PCL-5 avoidance	T1	2.71	0.56	2.70	0.55	0.01(−0.91*to*0.94)	0.98
	T2	2.64	0.56	2.18	0.55	0.45(−0.47*to*1.38)	0.33
	T3	2.66	0.56	2.27	0.55	0.39(−0.53*to*1.32)	0.40
PCL-5 cognitive and mood	T1	8.35	1.55	8.68	1.53	−0.33(−2.83*to*2.18)	0.80
	T2	8.39	1.55	8.48	1.53	−0.09(−2.6*to*2.41)	0.94
	T3	6.95	1.55	6.47	1.53	0.48(−2.02*to*2.99)	0.70
PCL-5 arousal and reactivity	T1	8.51	1.27	8.80	1.26	−0.29(−2.41*to*1.84)	0.79
	T2	8.80	1.27	8.21	1.26	0.59(−1.54*to*2.72)	0.58
	T3	6.93	1.27	6.24	1.26	0.7(−1.43*to*2.82)	0.52
HSCL-25 total	T1	31.20	5.54	30.90	5.46	0.29(−8.74*to*9.31)	0.95
	T2	30.30	5.54	28.40	5.46	1.88(−7.14*to*10.91)	0.68
	T3	26.20	5.54	25.80	5.46	0.4(−8.63*to*9.42)	0.93
HSCL-25 anxiety	T1	14.40	2.31	13.40	2.28	0.93(−2.85*to*4.7)	0.63
	T2	14.70	2.31	13.40	2.28	1.32(−2.46*to*5.09)	0.49
	T3	12.50	2.31	11.00	2.28	1.52(−2.26*to*5.3)	0.43
HSCL-25 depression	T1	16.70	3.42	15.50	3.38	−0.49(−6.09*to*5.12)	0.86
	T2	16.50	3.42	15.50	3.38	0.98(−4.63*to*6.58)	0.73
	T3	13.60	3.42	15.60	3.38	−1.97(−7.58*to*3.64)	0.49
WHOQOL_total	T1	40.50	1.49	41.40	1.47	−0.87(−3.34*to*1.59)	0.48
	T2	41.90	1.49	40.80	1.47	1.15(−1.32*to*3.62)	0.36
	T3	42.50	1.49	42.30	1.47	0.22(−2.25*to*2.69)	0.86

*Bonferroni correction-significant, p < 0.005.

*PCL-5; PTSD Checklist for DSM-5, HSCL-25; the Hopkins Symptom Checklist-25, WHOQoL BREF; the Brief version World Health Organization Quality of Life, SD, Standard Deviation.*

#### Depression and Anxiety Symptoms

There was a main effect of time for HSCL-25 total scores. The results indicate a significant reduction in HSCL-25 total scores from baseline to T3 for both groups (*p* < 0.001). *Post-hoc* tests showed HSCL-25 total scores were lower at T1, T2, and T3 than T0 in both groups (Bonferroni-adjusted *p* < 0.005). HSCL-25 total scores dropped by a clinically significant average of 42.30 points at 3 months after treatment (T3; *p* < 0.001). There was no effect of group (*p* = 0.72) nor of group-time interactions (*p* = 0.66) on HSCL-25 scores. No significant differences between the EMD and retrieval-only groups were found from baseline to T3 on HSCL-25 total scores. Furthermore, there was no significant difference between retrieval-only and EMD groups on all subscales of HSCL-25 score (Supplementary Appendix B). Per-protocol analysis of estimates for the effect of group, time, and group-time interaction for HSCL-25 indicated similar results (Supplementary Appendix C).

LMM in the intention to-treat sample for the HSCL-25 total score showed no significant differences between retrieval-only and EMD groups at T1 [*M* (*SE*) 31.20 (5.54) vs. 30.90 (5.46), 95% CI −8.74 to 9.31, *p* = 0.95], at T2 [*M* (*SE*) 30.30 (5.54) vs. 28.40 (5.46), 95% CI −7.14 to 10.91, *p* = 0.68], or at T3 [*M* (*SE*) 26.20 (5.54) vs. 25.80 (5.46), 95% CI −8.63 to 9.42, *p* = 0.93] (Table 2). Per-protocol analyses for HSCL-25 indicated similar results (Supplementary Appendix D).

#### Quality of Life

There was a main effect of time for WHOQoL-BREF total scores. The results showed a significant increase on WHOQoL-BREF total scores from baseline to T3 for both groups (*p* < 0.001). *Post-hoc* tests showed WHOQoL-BREF total scores were higher at T1, T2, and T3 than T0 in both groups (Bonferroni-adjusted *p* < 0.005). WHOQoL-BREF total scores increased by a clinically significant average of 6.40 points at 3-months after treatment (T3; *p* < 0.001). We found that there was no effect of group (*p* = 0.57) nor of group-time interaction (*p* = 0.48) on WHOQoL-BREF scores. No significant differences between the EMD and retrieval-only groups were found from baseline to T3 on WHOQoL-BREF scores. Similarly, none of the WHOQoL-BREF dimensions was significantly different between retrieval-only and EMD groups at T1, T2, and T3 (Supplementary Appendix B). Per-protocol analysis of estimates for the effect of group, time, and group-time interaction for WHOQoL-BREF indicated similar results (Supplementary Appendix C).

The LMM analysis showed no significant differences in WHOQoL total scores between retrieval-only and EMD groups at T1 [*M* (*SE*) 40.50 (1.49) vs. 41.40 (1.47), 95% CI −3.34 to 1.59, *p* = 0.48], at T2 [*M* (*SE*) 41.90 (1.49) vs. 40.80 (1.47), 95% CI −1.32 to 3.62, *p* = 0.36], or T3 [*M* (*SE*) 42.50 (1.49) vs. 42.30 (1.47), 95% CI −2.25 to 2.69, *p* = 0.86] (Table 2). Per-protocol analyses [*n* (T1) = 84, *n* (T2) = 82, *n* (T3) = 69] indicated similar results (Supplementary Appendix D).

## Discussion

The primary aim of the current study was to evaluate the effectiveness of EMD in reducing PTSD symptoms compared to a retrieval-only control condition among Indonesian adults diagnosed with PTSD. As a secondary aim, it was also examined whether EMD compared to retrieval-only was related to reductions in depression or anxiety symptoms, and improvement of quality of life scores. This study showed that both EMD and retrieval-only reduced PTSD, depression or anxiety, and improved quality of life after treatment and over the course of follow-up of 3-months, but no significant differences between the EMD and retrieval-only conditions were found. Thus, our hypothesis that EMs would be associated with stronger reductions in symptoms of PTSD, anxiety or depression and improvements in quality of life than retrieval only was not supported.

Our results are not in line with a previous dismantling study on EMDR (Sack et al., 2016). The study of Sack et al. (2016) found that EM as a dual-task had no additional treatment effects compared to visually fixating a non-moving hand. When compared to the current study, there are some clear differences with the study design of Sack et al. (2016). According to Sack et al. (2016), fixation on a non-moving hand of the therapist as a dual task generates a dual focus of attention that might help reduce associated PTSD symptoms. In our study, we used a retrieval-only condition and not another dual task condition, to compare with the eye moment element of EMDR. Despite these differences in comparison conditions, both Sack et al. (2016) and the current study reported no additional effects of EM on reduction of PTSD symptoms.

The results of current study are also in contrast with findings from studies described in the meta-analysis (Lee and Cuijpers, 2013), showing an overall moderate effect for additive EM across EMDR studies. The fact that we did not find a difference between EMD and the retrieval-only control condition, is perhaps due to the usage of an active control condition instead of a non-active control condition. Furthermore, Lee and Cuijpers (2013) reported that only six of the fourteen included treatment studies investigated the effects of EM with participants who had a DSM diagnosis of PTSD, while the other eight studies focused on students who had distressing memory or anxiety. Indeed, these six included studies showed that EMDR was superior to control, but four out of these studies found no significant difference between an EMDR and a control condition with a similar procedure but without eye movements (exposure only) in reducing PTSD symptoms or SUD scale (Sanderson and Carpenter, 1992; Renfrey and Richard Spates, 1994; Devilly et al., 1998; Devilly, 2002). These methodological issues regarding the usage of control conditions in the studies reviews by Lee and Cuijpers (2013) may explain why the findings reported in the meta-analysis indicated a significant difference between EM and control conditions, in contrast to the results of our study. It should also be noted that the quality of the included studies in this meta-analysis (Lee and Cuijpers, 2013) was low, which may have distorted the outcome. Our results are also in contrast with the results of experimental studies, which have provided strong evidence of the effects of EM in reducing vividness of intrusive images and emotionality (van den Hout and Engelhard, 2012; Landin-Romero et al., 2018; Houben et al., 2020; Mertens et al., 2021). It appears challenging to translate the results of experimental studies supporting the Working Memory model using healthy participants to estimate effects in clinical studies using clinical patients with standard EMDR procedures. The experimental studies usually examined emotionality and vividness of an autobiographical memory, which is clearly different from the broader picture of PTSD symptoms after real-life traumatic events (van Veen et al., 2019; Houben et al., 2020).

Although our study did not support the effect of EM element in EMD in reducing anxiety and depression, the current study confirmed that both EMD treatment and retrieval-on significantly reduced depression and anxiety symptoms after 3 months follow-up. The results of our study also add to the evidence that both EMD and retrieval-only therapy improve the quality of life of patients after treatment to 3 months follow-up. The current study, therefore, is in line with previous research that found EMDR to be an effective treatment for depressive or, trauma symptoms and improve the quality of life in depression patients with exposure to trauma (Cusack et al., 2016; Gauhar, 2016; Cuijpers et al., 2020).

On a final note, it should be considered that despite the sometimes convincing evidence supporting EMDR’s effectiveness for reducing PTSD symptoms (World Health Organization [WHO], 2013; Chen et al., 2014; Cusack et al., 2016; ISTSS, 2018; Wilson et al., 2018), some heterogeneity in methodology still exists (i.e., intervention, control condition, outcome measure, and follow-up procedures). The current study is different from other studies in control condition and the dose-response relationship (i.e., number of times treated and treatment duration) that may yield different results (Acarturk et al., 2016; Carletto et al., 2016; de Bont et al., 2016; Wilson et al., 2018).

### Strengths and Limitations of the Current Study

Strengths of the current study include the dismantling RCT design in which we compared single element of a multicomponent EMDR treatment. Further, despite challenges imposed by the COVID-19 pandemic, we were still able to include 90% of the original sample in our follow-up assessment.

Although efforts to lower biases were carried out, several limitations of this study should also be taken into account. We reached the determined sample size of *N* = 84 at posttreatment, since the attrition was lower than expected, but still the current study’s sample size can be considered relatively small for a study comparing two active treatments. In addition, not all instruments of the Bahasa Indonesian versions were validated for psychometric properties which may have compromised measurement validity (i.e., SCID-5). Furthermore, we cannot generalize the results to a population with a more equal gender distribution, because we included mostly female participants (91.2%). Finally, fidelity ratings should be included to assess protocol adherence.

### Clinical Implications

We have evaluated the effect of EM in EMD and retrieval-only groups in reducing PTSD symptoms. The results showed that both EMD and retrieval-only significantly reduced PTSD symptoms up to 3-months follow-up. In clinical practice, we may suggest that both EMDR and other retrieval-based PTSD treatments should be part of trauma professionals ’toolkits. Unfortunately, there is a large gap between the number of people in need of effective treatments for mental health problems such as PTSD, and people who are receiving them. This is especially true in a low and middle income country such as Indonesia (World Health Organization, 2012). In consideration that Indonesia is a country strongly calling for efficient and effective treatment methods that can be easily and widely disseminated, EMDR is an important tool in meeting this surging clinical demand.

### Research Implications

First, future studies are advised to include larger clinical samples. Further, measurements can be complemented by other tools such as eye-tracking software, which functions to identify and monitor a person’s visual attention in terms of location, objects, and duration. Thus, this software may provide input on which form of EM is occurring and whether have an effect on the treatment’s effectiveness. It may also be worthwhile to examine potential underlying neurocognitive and psychobiological mechanisms (Susanty et al., 2021b) that may be involved in the effects of retrieval-based therapies, including EMDR, on PTSD symptoms reduction.

## Conclusion

Our study did not support the idea that the EM component in EMDR has a significant effect over retrieval-only in reducing PTSD symptoms. Therefore, we conclude that both EMDR and retrieval only can be used to reduce PTSD symptoms. These findings not only conflict with the considerable body of literature that has demonstrated the efficacy of the EM component, but also did not provide further support for the working memory theory stating that dual tasks are more effective that retrieval only in reducing emotionality and vividness of traumatic memories. Further research to identify the exact mechanisms of EMDR’s effective treatment elements in clinical samples is needed.

## Data Availability Statement

The raw data supporting the conclusions of this article will be made available by the authors, without undue reservation.

## Ethics Statement

The studies involving human participants were reviewed and approved by the Research Ethics Committee of Universitas Padjadjaran Bandung on 2 July 2018 (Document number: 3 35/UN6.KEP/EC/2018). The patients/participants provided their written informed consent to participate in this study.

## Author Contributions

ES, MS, and AH worked on the original idea of this study and developed the design. ES prepared the manuscript. MS and AH provided detailed feedback and input on all aspects of the manuscript. WS gave input on methodology and ethical issues. YS conducted the quantitative analysis. All authors contributed to this trial study and approved of the final version of the manuscript.

## Conflict of Interest

The authors declare that the research was conducted in the absence of any commercial or financial relationships that could be construed as a potential conflict of interest.

## Publisher’s Note

All claims expressed in this article are solely those of the authors and do not necessarily represent those of their affiliated organizations, or those of the publisher, the editors and the reviewers. Any product that may be evaluated in this article, or claim that may be made by its manufacturer, is not guaranteed or endorsed by the publisher.

## Footnotes

1
www.castoredc.com
