# Supplementary material for: The Effectiveness of Eye Movement Desensitization for Post-traumatic Stress Disorder in Indonesia: A Randomized Controlled Trial
Source: Front Psychol. 2022 Apr 25;13:845520. doi: 10.3389/fpsyg.2022.845520 (PMC9081880; doi:10.3389/fpsyg.2022.845520)
Supplement: Supplementary file 2 [file Table_2.docx]

Appendix B. Estimate for the effect of group, time, and group-time interaction (intention to treat, N =91)

| **Variable** | **Time** | | | | | | | | | | | | | | | | | **Group-time interaction** | | | | | | | | | | | | | | | | | |  |
| --- | --- | --- | --- | --- | --- | --- | --- | --- | --- | --- | --- | --- | --- | --- | --- | --- | --- | --- | --- | --- | --- | --- | --- | --- | --- | --- | --- | --- | --- | --- | --- | --- | --- | --- | --- | --- |
|  | **T1** | | | **T2** | | | | | | | **T3** | | | | | | | **T1** | | | | | | **T2** | | | | | | **T3** | | | | | |  |
|  | **β** | **SE** | ***p* -value** | | | **β** | | **SE** | | ***p* -value** | | | **β** | | **SE** | | ***p* -value** | | **β** | | **SE** | | ***p* -value** | | **β** | | **SE** | | ***p* -value** | | **β** | | **SE** | | ***p* -value** | |
| **PCL-5** |  |  |  | |  | |  | |  | | |  | |  | |  | |  | |  | |  | |  | |  | |  | |  | |  | |  | |  |
| Intrusive | -9.16 | 0.64 | 0.00 | | -9.38 | | 0.64 | | 0.00 | | | -10.45 | | 0.64 | | 0.00 | | -0.42 | | 0.89 | | 0.64 | | -0.92 | | 0.89 | | 0.30 | | -0.26 | | 0.89 | | 0.77 | |  |
| Avoidance | -3.11 | 0.35 | 0.00 | | -3.18 | | 0.35 | | 0.00 | | | -3.16 | | 0.35 | | 0.00 | | -0.40 | | 0.49 | | 0.42 | | -0.84 | | 0.49 | | 0.09 | | -0.78 | | 0.49 | | 0.11 | |  |
| Cognitive and mood | -12.16 | 0.93 | 0.00 | | -12.12 | | 0.93 | | 0.00 | | | -13.56 | | 0.93 | | 0.00 | | -0.12 | | 1.30 | | 0.93 | | -0.36 | | 1.30 | | 0.78 | | -0.93 | | 1.30 | | 0.47 | |  |
| Arousal and reactivity | -9.77 | 0.83 | 0.00 | | -9.49 | | 0.83 | | 0.00 | | | -11.36 | | 0.83 | | 0.00 | | -0.51 | | 1.15 | | 0.66 | | -0.36 | | 1.15 | | 0.75 | | -0.47 | | 1.15 | | 0.68 | |  |
| PCL-5 total | -32.18 | 2.99 | 0.00 | | -33.05 | | 2.99 | | 0.00 | | | -23.10 | | 2.99 | | 0.00 | | -3.00 | | 4.17 | | 0.47 | | -3.05 | | 4.17 | | 0.47 | | -4.78 | | 4.17 | | 0.25 | |  |
| **HSCL-25** |  |  |  | |  | |  | |  | | |  | |  | |  | |  | |  | |  | |  | |  | |  | |  | |  | |  | |  |
| Anxiety | -14.74 | 1.43 | 0.00 | | -14.40 | | 1.43 | | 0.00 | | | -16.60 | | 1.43 | | 0.00 | | -1.84 | | 1.98 | | 0.35 | | -2.23 | | 1.98 | | 0.26 | | -2.43 | | 1.98 | | 0.22 | |  |
| Depression | -22.73 | 2.10 | 0.00 | | -22.91 | | 2.10 | | 0.00 | | | -25.79 | | 2.1 | | 0.00 | | -0.27 | | 2.92 | | 0.92 | | -1.73 | | 2.93 | | 0.55 | | 1.22 | | 2.93 | | 0.68 | |  |
| HSCL-25 total | -37.27 | 3.35 | 0.00 | | -38.19 | | 3.35 | | 0.00 | | | -42.30 | | 3.35 | | 0.00 | | -1.94 | | 4.67 | | 0.67 | | -3.54 | | 4.67 | | 0.45 | | -2.06 | | 4.68 | | 0.66 | |  |
| **WHOQoL** |  |  |  | |  | |  | |  | | |  | |  | |  | |  | |  | |  | |  | |  | |  | |  | |  | |  | |  |
| Physical | 0.63 | 0.25 | 0.00 | | 0.58 | | 0.25 | | 0.01 | | | 0.75 | | 0.25 | | 0.00 | | -0.11 | | 0.35 | | 0.75 | | -0.48 | | 0.35 | | 0.16 | | -0.16 | | 0.35 | | 0.64 | |  |
| Psychological | 1.72 | 0.27 | 0.00 | | 1.39 | | 0.27 | | 0.02 | | | 1.75 | | 0.27 | | 0.00 | | -0.60 | | 0.38 | | 0.11 | | -0.22 | | 0.38 | | 0.56 | | -0.06 | | 0.38 | | 0.88 | |  |
| Social | 2.09 | 0.60 | 0.00 | | 2.61 | | 0.60 | | 0.00 | | | 3.33 | | 0.6 | | 0.00 | | -0.51 | | 0.84 | | 0.55 | | -0.44 | | 0.84 | | 0.60 | | -1.20 | | 0.84 | | 0.16 | |  |
| Environmental | 0.38 | 0.31 | 0.22 | | 0.37 | | 0.31 | | 0.23 | | | 0.55 | | 0.32 | | 0.08 | | 0.42 | | 0.43 | | 0.33 | | -0.03 | | 0.43 | | 0.95 | | 0.48 | | 0.43 | | 0.26 | |  |
| WHOQoL | 4.41 | 0.95 | 0.00 | | 5.80 | | 0.95 | | 0.00 | | | 6.40 | | 0.95 | | 0.00 | | 0.16 | | 1.32 | | 0.90 | | -1.87 | | 1.32 | | 0.16 | | -0.94 | | 1.32 | | 0.48 | |  |

Notes:

SE = Standard error, HSCL-25 = the Hopkins Symptom Checklist- 25, PCL-5 = PTSD Checklist for DSM-5 , T1 = time point at a week after treatment session, T2 = time point at one month after treatment session , T3 = 3-month after treatment session, WHOQoL-BREF = World Health Organization Quality of Life Scale
