# Supplementary material for: The Effectiveness of Eye Movement Desensitization for Post-traumatic Stress Disorder in Indonesia: A Randomized Controlled Trial
Source: Front Psychol. 2022 Apr 25;13:845520. doi: 10.3389/fpsyg.2022.845520 (PMC9081880; doi:10.3389/fpsyg.2022.845520)
Supplement: Supplementary file 3 [file Table_3.docx]

Appendix C. Estimates for the effect of group, time, and group-time interaction (per protocol, N (T1)= 84, N (T2)= 82, N (T3) = 69)

| **Variable** | **Time** | | | | | | | | | | | **Group-time interaction** | | | | | | | | | | | |  |
| --- | --- | --- | --- | --- | --- | --- | --- | --- | --- | --- | --- | --- | --- | --- | --- | --- | --- | --- | --- | --- | --- | --- | --- | --- |
|  | **T1** | | | **T2** | | | | **T3** | | | | **T1** | | | | **T2** | | | | **T3** | | | |  |
|  | **β** | **SE** | ***p* -value** | | **β** | **SE** | ***p* -value** | | **β** | **SE** | ***p* -value** | | **β** | **SE** | ***p* -value** | | **β** | **SE** | ***p* -value** | | **β** | **SE** | ***p*-value** | |
| **PCL-5** |  |  |  | |  |  |  | |  |  |  | |  |  |  | |  |  |  | |  |  |  | |
| PCL-5 total | -34.70 | 3.04 | 0.00 | | -34.52 | 3.04 | 0.00 | | -22.55 | 3.04 | 0.00 | | -4.82 | 4.37 | 0.27 | | -6.07 | 4.37 | 0.17 | | -5.97 | 4.37 | 0.17 | |
| Intrusive | -9.55 | 0.62 | 0.00 | | -9.55 | 0.62 | 0.00 | | -10.30 | 0.62 | 0.00 | | -1.49 | 0.90 | 0.10 | | -1.94 | 0.90 | 0.03 | | -0.86 | 0.90 | 0.34 | |
| Avoidance | -2.97 | 0.37 | 0.00 | | -3.18 | 0.37 | 0.00 | | 0.30 | 0.37 | 0.00 | | -1.42 | 0.53 | 0.01 | | -1.63 | 0.53 | 0.00 | | -1.29 | 0.53 | 0.02 | |
| Cognitive and mood | -12.39 | 0.90 | 0.00 | | -12.15 | 0.90 | 0.00 | | -12.85 | 0.9 | 0.00 | | -1.12 | 1.29 | 0.39 | | -1.46 | 1.29 | 0.26 | | -1.15 | 1.29 | 0.37 | |
| Arousal and reactivity | -9.79 | 0.83 | 0.00 | | -9.64 | 0.83 | 0.00 | | -10.49 | 0.83 | 0.00 | | -0.79 | 1.19 | 0.51 | | -1.04 | 1.19 | 0.38 | | -0.87 | 1.19 | 0.47 | |
| **HSCL-25** |  |  |  | |  |  |  | |  |  |  | |  |  |  | |  |  |  | |  |  |  | |
| HSCL-25 total | -39.03 | 3.40 | 0.00 | | -41.15 | 3.40 | 0.00 | | -42.06 | 3.40 | 0.00 | | -8.39 | 4.89 | 0.09 | | -6.17 | 4.89 | 0.21 | | -3.20 | 4.87 | 0.51 | |
| Anxiety | -15.39 | 1.44 | 0.00 | | -16.12 | 1.44 | 0.00 | | -16.46 | 1.44 | 0.00 | | -3.64 | 2.07 | 0.08 | | -2.72 | 2.07 | 0.19 | | -2.51 | 2.07 | 0.23 | |
| Depression | -23.64 | 2.18 | 0.00 | | -25.03 | 2.18 | 0.00 | | -25.61 | 2.18 | 0.00 | | -4.75 | 3.13 | 0.13 | | -3.45 | 3.13 | 0.27 | | -0.68 | 3.13 | 0.83 | |
| **WHOQoL** |  |  |  | |  |  |  | |  |  |  | |  |  |  | |  |  |  | |  |  |  | |
| WHOQoL total | 4.36 | 0.98 | 0.00 | | 5.64 | 0.98 | 0.00 | | 5.94 | 0.98 | 0.00 | | 0.19 | 1.41 | 0.90 | | -0.83 | 1.41 | 0.56 | | -0.29 | 1.41 | 0.84 | |
| Physical | 0.61 | 0.24 | 0.12 | | 0.79 | 0.24 | 0.00 | | 0.94 | 0.24 | 0.00 | | -0.15 | 0.35 | 0.01 | | -0.30 | 0.35 | 0.66 | | -0.23 | 0.35 | 0.51 | |
| Psychological | 1.73 | 0.29 | 0.00 | | 1.73 | 0.29 | 0.00 | | 1.88 | 0.29 | 0.00 | | -0.47 | 0.42 | 0.26 | | -0.44 | 0.42 | 0.29 | | -0.43 | 0.42 | 0.30 | |
| Social | 1.67 | 0.65 | 0.01 | | 2.39 | 0.65 | 0.00 | | 2.49 | 0.65 | 0.00 | | 0.20 | 0.93 | 0.83 | | -0.20 | 0.93 | 0.83 | | -0.39 | 0.93 | 0.68 | |
| Environmental | 0.36 | 0.25 | 0.22 | | 0.73 | 0.25 | 0.23 | | 0.64 | 0.25 | 0.01 | | 0.60 | 0.36 | 0.09 | | 0.11 | 0.36 | 0.76 | | 0.75 | 0.36 | 0.04 | |

Notes:

SE = Standard error, HSCL-25 = the Hopkins Symptom Checklist- 25, PCL-5 = PTSD Checklist for DSM-5 , T1 = time point at a week after treatment session, T2 = time point at one month after treatment session , T3 = 3-month after treatment session, WHOQoL-BREF = World Health Organization Quality of Life Scale
