# Supplementary material for: The Effectiveness of Eye Movement Desensitization for Post-traumatic Stress Disorder in Indonesia: A Randomized Controlled Trial
Source: Front Psychol. 2022 Apr 25;13:845520. doi: 10.3389/fpsyg.2022.845520 (PMC9081880; doi:10.3389/fpsyg.2022.845520)
Supplement: Supplementary file 4 [file Table_4.docx]

| Appendix D. Summary statistics and results from mixed-model analysis for symptoms of PTSD, anxiety, depression s and quality of life (per protocol, N (T1)= 84, N (T2)= 82, N (T3) = 69) | | | | | | | |
| --- | --- | --- | --- | --- | --- | --- | --- |
| **Outcomes** | **Measurement time** | **Mean (SD)** | | | | **Mean difference (95% confidence interval)** | ***p*-value *** |
|  |  | **Retrieval only** | | **EMD** | |  |  |
| PCL-5 total | T1 | 30.00 | 4.95 | 25.30 | 4.99 | 4.75(-4.16 to 13.65) | 0.29 |
|  | T2 | 30.20 | 4.95 | 24.20 | 4.99 | 5.99 (-2.91 to 14.90) | 0.19 |
|  | T3 | 42.20 | 4.95 | 36.30 | 4.99 | 5.90 (-3.01 to 14.81) | 0.19 |
| PCL-5 Intrusion | T1 | 7.52 | 1.08 | 5.92 | 1.09 | 1.60 (-0.31 to 3.51) | 0.10 |
|  | T2 | 7.52 | 1.08 | 5.47 | 1.09 | 2.05 (0.14 to 3.96) | 0.04 |
|  | T3 | 6.77 | 1.08 | 5.79 | 1.09 | 0.97 (-0.94 to 2.88) | 0.32 |
| PCL-5 Avoidance | T1 | 3.15 | 0.54 | 2.36 | 0.55 | 0.79 (-0.21 to 1.79) | 0.12 |
|  | T2 | 2.94 | 0.54 | 1.94 | 0.55 | 1.00 (0.00 to 2.00) | 0.05 |
|  | T3 | 3.12 | 0.54 | 2.46 | 0.55 | 0.67 (-0.33 to 1.67) | 0.19 |
| PCL-5 Cognitive and mood | T1 | 8.95 | 1.49 | 7.85 | 1.50 | 1.10 (-1.56 to 3.77) | 0.42 |
|  | T2 | 9.19 | 1.49 | 7.75 | 1.50 | 1.44 (-1.22 to 4.11) | 0.29 |
|  | T3 | 8.49 | 1.49 | 7.36 | 1.50 | 1.13 (-1.53 to 3.80) | 0.40 |
| PCL-5 Arousal and reactivity | T1 | 9.11 | 1.26 | 7.99 | 1.27 | 1.12 (-1.19 to 3.43) | 0.34 |
|  | T2 | 9.26 | 1.26 | 7.89 | 1.27 | 1.37 (-0.94 to 3.68) | 0.24 |
|  | T3 | 8.41 | 1.26 | 7.21 | 1.27 | 1.20 (-1.11 to 3.51) | 0.31 |
| HSCL-25 total | T1 | 33.20 | 5.38 | 26.80 | 5.42 | 6.38 (-3.36 to 16.12) | 0.20 |
|  | T2 | 31.10 | 5.38 | 26.90 | 5.42 | 4.17 (-5.57 to 13.91) | 0.40 |
|  | T3 | 30.20 | 5.38 | 29.00 | 5.42 | 1.19 (-8.55 to 10.93) | 0.81 |
| HSCL-25 Anxiety | T1 | 15.60 | 2.37 | 12.60 | 2.38 | 3.06 (-1.17 to 7.30) | 0.16 |
|  | T2 | 14.90 | 2.37 | 12.80 | 2.38 | 2.14 (-2.09 to 6.37) | 0.32 |
|  | T3 | 14.60 | 2.37 | 12.60 | 2.38 | 1.94 (-2.30 to 6.17) | 0.37 |
| HSCL-25 Depression | T1 | 17.60 | 3.22 | 14.30 | 3.25 | 3.32 (-2.62 to 9.27) | 0.27 |
|  | T2 | 16.20 | 3.22 | 14.20 | 3.25 | 2.03 (-3.92 to 7.97) | 0.50 |
|  | T3 | 15.60 | 3.22 | 16.40 | 3.25 | -0.74 (-6.69 to 5.20) | 0.81 |
| WHOQOL_total | T1 | 40.10 | 1.57 | 40.7 | 1.59 | -0.63 (-3.47 to 2.21) | 0.66 |
|  | T2 | 41.30 | 1.57 | 40.9 | 1.59 | 0.38 (-2.46 to 3.23) | 0.79 |
|  | T3 | 41.60 | 1.57 | 41.8 | 1.59 | -0.15 (-3.00 to 2.69) | 0.92 |

Notes:

*Bonferroni correction-significant, *p* < .005

PCL-5; PTSD Checklist for DSM-5t, HSCL-25; the Hopkins Symptom Checklist-25, WHOQoL; the World Health Organization Quality of Life, SD = Standard Deviation
